# Genotypic characterization of antibiotic resistance genes in multidrug-resistant *Staphylococcus aureus* isolated from companion and livestock animals in Indonesia

**DOI:** 10.14202/vetworld.2026.978-991

**Published:** 2026-03-15

**Authors:** Alyaa Rifqoh Putri Yosyana, Siti Isrina Oktavia Salasia, Ghias Ghifari Alhadz, Fatkhanuddin Aziz

**Affiliations:** 1 Department of Clinical Pathology, Faculty of Veterinary Medicine, Universitas Gadjah Mada, Yogyakarta, Indonesia; 2 Department of Bioresources Technology and Veterinary, Vocational College, Universitas Gadjah Mada, Yogyakarta, Indonesia

**Keywords:** antimicrobial resistance, companion animals, Indonesia, livestock animals, multidrug resistance, One Health, *Staphylococcus aureus*, zoonotic transmission

## Abstract

**Background and Aim:**

Antimicrobial resistance (AMR) in *Staphylococcus aureus* represents a critical threat to veterinary and public health, with multidrug-resistant (MDR) strains facilitating zoonotic transmission across animal species. This study aimed to investigate the prevalence and diversity of key antibiotic resistance genes in MDR *S. aureus* isolates from companion and livestock animals in Indonesia, and to assess their potential for interspecies dissemination within a One Health framework.

**Materials and Methods:**

A total of 121 bacterial isolates were collected from bovine milk (n = 30), cats (n = 61), dogs (n = 18), rabbits (n = 7), and goats (n = 5) between June 2024 and August 2025 in Yogyakarta and Central Java, Indonesia. Phenotypic identification involved biochemical tests (catalase, coagulase, mannitol fermentation), antimicrobial susceptibility testing via disk diffusion against seven antibiotics (tetracycline, gentamicin, erythromycin, penicillin G, cefoxitin, ciprofloxacin, clindamycin), and genotypic confirmation using polymerase chain reaction (PCR) for *23S rRNA*, *nuc*, and *coa* genes. Resistance genes (*mecA*, *blaZ*, *aacA-D*, *ermA*, *tetK*, *tetM*, *msrB*, *linA*, *norA*) were detected via targeted PCR. MDR was defined as resistance to ≥ 3 antimicrobial classes. Statistical analysis included Fisher’s exact test (p < 0.05) for comparing resistance patterns across hosts.

**Results:**

Of the isolates, 55 (45.5%) were confirmed as *S. aureus*, with the highest prevalence in bovine milk (80%) and rabbits (85.7%). All exhibited MDR phenotypes, predominantly to penicillin (20%–72%), tetracycline (17%–28%), and clindamycin. Erythromycin resistance varied significantly across sources (p < 0.05). Genotypically, *tetK* was universal (100%), followed by *linA* (85.5%), *norA* (81.8%), *mecA* (76.4%), and *blaZ* (69.1%). Significant differences (p < 0.05) occurred in *tetM*, *blaZ*, *aacA-D*, *norA*, and *msrB* distribution. Co-occurrence of *mecA*, *blaZ*, and *tetK* suggested horizontal gene transfer. Phenotypic-genotypic discrepancies were noted, potentially due to alternative genes (e.g., *mecC*, *ermC*) or regulatory mechanisms. MDR patterns were prominent in bovine and cat isolates, with complex gene combinations (≥ 4 genes) in over 50% of cases.

**Conclusion:**

This study reveals shared resistance gene profiles in MDR *S. aureus* from Indonesian animals, highlighting zoonotic risks and the need for integrated AMR surveillance. Limitations include the targeted PCR’s scope; future work should employ whole-genome sequencing to enable comprehensive resistome analysis and transmission studies.

## INTRODUCTION

Antimicrobial resistance (AMR) has become a serious global health threat, affecting human and veterinary medicine. Pathogenic bacteria continue to evolve resistance mechanisms to multiple antibiotics, reducing treatment effectiveness and increasing morbidity and mortality from bacterial infections. The scarcity of new antimicrobial discoveries intensifies the issue of replacing ineffective drugs. In 2019, the World Health Organization (WHO) estimated that 1.2 million deaths were directly attributable to antibiotic-resistant bacterial infections in 2019, and that up to 4.95 million deaths were associated with antimicrobial resistance in 2022. Without effective interventions, AMR is projected to cause 10 million deaths annually by 2050 [1, 2].

The emergence of multidrug-resistant (MDR) bacteria, defined as strains resistant to three or more antimicrobial classes, poses a significant challenge for infection control. MDR bacteria have been detected not only in companion animals but also in livestock and animal-derived food products, such as milk, meat, and processed animal-derived foods [3, 4]. Their presence in animals poses a zoonotic risk, as transmission can occur through direct contact or the food chain. Among these bacteria, *Staphylococcus aureus*, including its methicillin-resistant variant (methicillin-resistant *S. aureus* [MRSA]), is a major pathogen associated with AMR and MDR infections.

The MDR of *S. aureus* has been widely reported in animal populations across different regions. Studies from China have reported high resistance rates to penicillin, erythromycin, tetracycline, and clindamycin among *S. aureus* isolates from animal-derived food products [5]. *S. aureus* isolated from dairy cattle in Central Java and Riau showed substantial resistance to ampicillin, oxacillin, tetracycline, and erythromycin in Indonesia [6]. In Malaysia, *S. aureus* isolated from stray cats exhibited resistance to multiple critical antimicrobials [7]. These findings highlight the widespread distribution of MDR *S. aureus* in animal sources and its potential role in the dissemination of resistance across species.

Specific resistance genes mediate antibiotic resistance in *S. aureus*. The *mecA* gene confers methicillin resistance, *blaZ* encodes β-lactamase, conferring penicillin resistance, *aacA-D* is associated with aminoglycoside resistance, *tetK* and *tetM* confer tetracycline resistance, and *ermA*, *ermB*, and *ermC* mediate macrolide-lincosamide-streptogramin B (MLSB) resistance [8]. Previous studies have reported that *S. aureus* isolates from animals carrying multiple resistance genes, including *mecA*, are resistant to ampicillin, oxacillin, tetracycline, penicillin G, and erythromycin [9].

Despite increasing reports of MDR *S. aureus* in animals, data on the genetic determinants of AMR in *S. aureus* isolated from diverse animal sources in Indonesia remain scarce, particularly studies that simultaneously assess multiple resistance genes and their potential role in interspecies transmission. Most available studies have focused primarily on phenotypic resistance or on a limited set of host species, leaving a critical gap in understanding the molecular epidemiology of MDR *S. aureus* in the animal sector. In Indonesia, close interactions among livestock, companion animals, humans, and the environment facilitate the circulation of antimicrobial-resistant *S. aureus*. Animals may serve as reservoirs and transmission bridges for resistant strains at this One Health interface, underscoring the importance of characterizing resistance gene profiles across animal sources to support integrated AMR surveillance and control.

Addressing this gap is essential to support national and global AMR surveillance initiatives in line with the WHO Global Action Plan on AMR [10]. Therefore, this study aimed to characterize the genotypes of key antimicrobial resistance genes in multidrug-resistant *S. aureus* isolates from animals in Yogyakarta and Central Java, Indonesia, to provide insight into the distribution of resistance genes and inform the risk assessment of zoonotic and interspecies dissemination of AMR. Yogyakarta and Central Java were selected as study sites due to their high livestock production density, substantial companion animal populations, and frequent human–animal contact, making these regions representative and relevant settings for monitoring the circulation of antimicrobial-resistant *S. aureus* at the animal–human interface.

## MATERIALS AND METHODS

### Ethical approval

Informed consent and permission for sample collection were obtained from animal owners and veterinary facilities before sampling. The Research Ethics Committee of the Faculty of Veterinary Medicine, Universitas Gadjah Mada, Yogyakarta, Indonesia, approved the collection and use of animal isolates (Approval No. 143/EC-FKH/Int./2024).

### Study design, period, and location

This study used a cross-sectional, laboratory-based observational design to investigate the genotypic characteristics of AMR in *S. aureus* isolated from animals. Sampling was conducted from June 2024 to August 2025. Laboratory analyses were performed at the Clinical Pathology Laboratory, Faculty of Veterinary Medicine, and the Animal Science Learning Center at Universitas Gadjah Mada in Yogyakarta, Indonesia.

### Sample collection and bacterial isolation

A total of 121 bacterial isolates, comprising 30 from bovine milk, 61 from cats, 18 from dogs, 7 from rabbits, and 5 from goat milk, were used in this study. Bovine and goat milk samples were obtained from dairy farms in Boyolali, Central Java, Indonesia, whereas companion animal samples were collected from veterinary clinics and hospitals in Yogyakarta and Semarang.

Animals exhibiting clinical signs suggestive of bacterial infection, such as pyogenic skin lesions, rhinitis, pyrexia, lethargy, or mastitis (dairy animals) were included in the study. Swab samples were collected from the oropharynx, nasal cavity, ears, and skin lesions of companion animals. Milk samples were aseptically collected from dairy animals with clinical or subclinical mastitis. Animals that had received systemic antibiotics within 2 weeks before sampling or samples showing mixed bacterial growth were excluded from the analysis.

Continuous sample collection was conducted throughout the study period to minimize seasonal or temporal bias. All samples were aseptically transported and processed immediately upon arrival at the laboratory. Specimens were cultured on 5% defibrinated sheep blood agar and incubated aerobically at 37°C for 24 h. Colonies with typical *S. aureus* morphology were selected for further identification by Gram staining, hemolytic activity testing, and subculture onto mannitol salt agar. Biochemical confirmation was performed using catalase and coagulase tests following standard protocols.

### Antimicrobial susceptibility testing

All *S. aureus* isolates were inoculated into tryptic soy broth (Merck, Germany) and incubated at 37°C for 24 h. The culture was washed with sterile phosphate-buffered saline (PBS) and diluted with sterile PBS to a turbidity equivalent to a 0.5 McFarland standard (HiMedia, USA). Susceptibility testing was performed using the Kirby–Bauer disk diffusion method in accordance with the Clinical and Laboratory Standards Institute (CLSI) guidelines (CLSI M100, 2024) [11].

The culture was standardized to a 0.5 McFarland standard, and 70 µL was spread on Mueller-Hinton agar (MHA; Merck, Germany) using a glass spreader. Antibiotic disks were placed on MHA at a specified distance from one another and incubated at 37°C for 24 h. The antibiotic disks (Oxoid, UK) used in this study were tetracycline (30 µg), gentamicin (10 µg), erythromycin (15 µg), penicillin G (10 µg), cefoxitin (30 µg), ciprofloxacin (5 µg), and clindamycin (10 µg). Inhibition zone diameters were measured and interpreted as susceptible, intermediate, or resistant according to CLSI M100 (2024) criteria. Multidrug resistance (MDR) was defined as resistance to ≥1 agent in ≥3 antimicrobial classes, as defined by Magiorakos *et al*. [12].

### DNA extraction, molecular identification, and resistance gene detection

Genomic DNA was extracted from *S. aureus* isolates using the Geneaid Bacteria DNA Kit (Geneaid, Taiwan) according to the manufacturer’s instructions. Molecular identification was performed by polymerase chain reaction (PCR) amplification of the *23S rRNA* and *nuc* genes, and the *coa* gene was amplified to characterize *S. aureus* isolates.

Detection of antibiotic resistance genes, including *mecA* (methicillin/oxacillin resistance), *blaZ* (penicillin G resistance), *aacA-D* (aminoglycoside resistance), *ermA*, *msrB*, and *linA* (macrolide-lincosamide-streptogramin B resistance), *tetK* and *tetM* (tetracycline resistance), and *norA* (ciprofloxacin resistance), was performed using specific primers and PCR conditions listed in Table 1. Each 10 μL PCR reaction mixture contained 0.5 μL of forward primer (10 pmol), 0.5 μL of reverse primer (10 pmol), 5 μL of PCR mix, 3.5 μL of nuclease-free water, and 0.5 μL of DNA template. MyTaq HS Red Mix (Bioline, UK) was used for the amplification of all target genes except *nuc*, for which PowerPol 2× PCR Mix with Dye (ABclonal, USA) was used. Briefly, the PCR tubes were centrifuged and placed in a thermal cycler under the corresponding conditions. All primers used in this study were selected from previously published studies and validated for specificity and sensitivity against *S. aureus* (Table 1) [13–21]. For quality assurance, each PCR run included a positive control consisting of DNA from a well-characterized *S. aureus* strain, previously confirmed phenotypically, genotypically, and by whole-genome sequencing, and a negative control containing nuclease-free water instead of template DNA. All PCR assays were performed in duplicate to ensure reproducibility, and consistent results were obtained across repeated runs.

**Table 1:** Oligonucleotide primers and polymerase chain reaction (PCR) programs for amplification of species-specific and antibiotic resistance genes in *Staphylococcus aureus*.

Target gene	Primer sequence (5’–3’)	Annealing (°C)	Target size (bp)	PCR program	Reference
*23S rRNA*	AGCGAGTTACAAAGGAGGAC; AGCTCAGCCTTAACGAGTAC	64	1250	35 cycles: 95°C 15 s/64°C 30 s/72°C 10 s	[9]
*nuc*	GCGATTGATGGTGATACGGTT; ACGCAAGCCTTGACGAACTAAAGC	55	279	37 cycles: 98°C 10 s/55°C 30 s/72°C 5 s	[9]
*coa*	GTCTTGAAAGTAGCTCATCTAAACTTG; ATCCAAATGTTCCATCGTTGTATTC	55	228	40 cycles: 95°C 10 s/55°C 20 s/72°C 25 s	[13]
*blaZ*	ACTTCAACACCTGCTGCTTTC; TGACCACTTTTATCAGCAACC	55	173	30 cycles: 94°C 30 s/55°C 30 s/72°C 1 min	[14]
*aacA-D*	TAATCCAAGAGCAATAAGGGC; GCCACACTATCATAACCACTA	55	227	30 cycles: 94°C 30 s/55°C 30 s/72°C 30 s	[15]
*ermA*	ACGATATTCACGGTTTACCCACTTA; AACCAGAAAAACCCTAAAGACACG	55	610	30 cycles: 94°C 30 s/55°C 30 s/72°C 30 s	[16]
*msrB*	TATGATATCCATAATAATTATCCAATC; AAGTTATATCATGAATAGATTGTCCTGTT	50	595	35 cycles: 95°C 15 s/50°C 15 s/72°C 10 s	[17]
*tetK*	GTGCGACAATAGGTAATAGT; GTAGTGACAATAAACCTCCTA	55	360	35 cycles: 95°C 30 s/55°C 30 s/72°C 10 s	[18]
*tetM*	AGTGGAGCGATTACAGAA; CATATGTCCTGGCGTGTCTA	55	158	35 cycles: 95°C 30 s/55°C 30 s/72°C 10 s	[19]
*linA*	GGTGGCTGGGGGGTAGATGTATTAACTGG; GCTTCTTTTGAAATACATGGTATTTTTCGATC	57	323	35 cycles: 95°C 15 s/57°C 15 s/72°C 10 s	[20]
*norA*	GACATTTCACCAAGCCATCAA; TGCCATAAATCCACCAATCC	55	102	30 cycles: 98°C 10 s/55°C 30 s/72°C 30 s	[21]
*mecA*	AAAATCGATGGTAAAGGTTGGC; AGTTCTGCAGTACCGGATTTGC	56	533	35 cycles: 95°C 30 s/55°C 30 s/72°C 10 s	[9]

The PCR products were separated by electrophoresis on a 1.5% (w/v) agarose gel (GeneDireX, USA) in 1× TBE buffer and stained with Safe-Red™ Gel Stain (ABM, Canada). Bands were visualized using a UV transilluminator and compared with a 100-bp DNA ladder (Smobio, Taiwan).

### Quality control strains

We ensured quality control by including a well-characterized *S. aureus* isolate that had been previously confirmed phenotypically and genotypically and further validated by whole-genome sequencing [9]. This isolate served as an internal positive control for biochemical identification, antimicrobial susceptibility testing, and PCR assays. These measures ensured the accuracy, reliability, and reproducibility of the phenotypic and molecular results.

### Statistical analysis

Descriptive statistics were used to summarize the distribution of *S. aureus* isolates and resistance genes across animal species (bovine milk, cats, dogs, rabbits, and goat milk). A comparative analysis of resistance gene prevalence and antimicrobial resistance patterns among host species was performed using Fisher’s exact test at a significance level of p < 0.05. The agreement between phenotypic AMR and the corresponding resistance genes was evaluated descriptively by assessing the concordance between disk diffusion results and PCR detection.

## RESULTS

### Identification of *S. aureus* isolates

A total of 55 isolates were confirmed as *S. aureus* based on colony morphology, biochemical characteristics, and PCR amplification of the *23S rRNA* and *nuc* genes. The isolates were obtained from various animal sources, including bovine milk (24/30), cats (18/61), dogs (5/18), rabbits (6/7), and goat milk (2/5).

### Phenotypic characteristics

**Figure 1:**
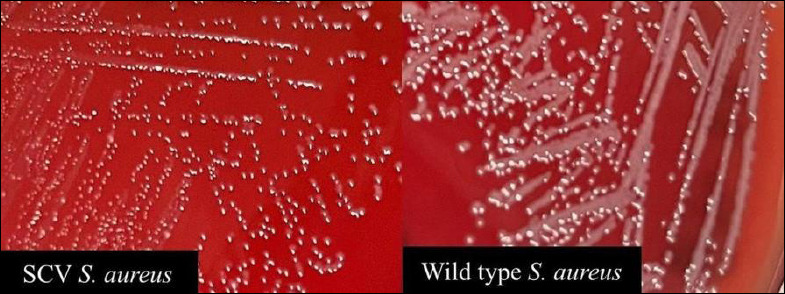
Comparison of colony morphology between small-colony variant and wild-type *Staphylococcus aureus* on blood agar.

Typical *S. aureus* colonies were round, smooth, convex, glistening, and grayish to golden-yellow on sheep blood agar (5%). Most isolates exhibited a creamy-to-opaque appearance with distinct hemolytic zones. All isolates were Gram-positive, catalase-positive cocci arranged in clusters. Mannitol fermentation was observed in 98.2% (54/55) of the isolates, and 43.6% (24/55) were coagulase-positive. Variable hemolysis patterns (α-, β-, and γ-hemolysis) were observed among the isolates obtained from different animal sources. In addition, 5 isolates from bovine milk (2/24, 8.3%), dog (2/5, 40%), and goat milk (1/2, 50%) exhibited atypical small-colony variant (SCV)-like morphology, characterized by pinpoint colonies smaller than those of wild-type *S. aureus* (Figure 1). These morphological variants were rare but consistently reproducible upon subculture. The phenotypic results were consistent with the general characteristics of *S. aureus* and supported the identification of the species before molecular analysis. Table 2 summarizes the detailed biochemical and hemolytic profiles.

**Table 2:** Phenotypic characteristics of *Staphylococcus aureus* isolates from various animal sources.

Source of the isolates	Number of samples	No. of identified *S. aureus*	Gram stain	Catalase-positive (%)	Positive mannitol fermentation (%)	Coagulase-positive (%)	Type of hemolysis observed*	SCV identified (%)
Bovine milk	30	24	+	83.3 (20/24)	100 (24/24)	33.3 (8/24)	α, β, γ	8.3 (2/24)
Cats	61	18	+	94.4 (17/18)	94.4 (17/18)	38.9 (7/18)	α, β, γ	0 (0/18)
Dogs	18	5	+	80.0 (4/5)	100 (5/5)	60.0 (3/5)	β, γ	40 (2/5)
Rabbits	7	6	+	83.3 (5/6)	100 (6/6)	83.3 (5/6)	β, γ	0 (0/6)
Goat milk	5	2	+	100 (2/2)	100 (2/2)	50.0 (1/2)	α, β	50 (1/2)
Total	121	55						

* Hemolysis types: α = Alpha-hemolysis, β = Beta-hemolysis, γ = Gamma-hemolysis (non-hemolytic), SCV = Small-colony variant

### Molecular confirmation

**Figure 2:**
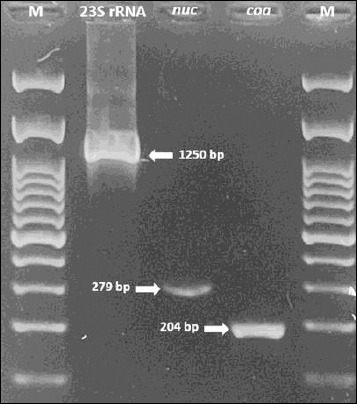
Polymerase chain reaction amplification of *23S rRNA* (1250 base pairs), *nuc* (279 base pairs), and *coa* (204 base pairs) genes in *Staphylococcus aureus* isolates. M: 100 base pairs of DNA ladder.

Molecular confirmation of all isolates was performed by PCR amplification of the *23S rRNA* and *nuc* genes. Characterization of *S. aureus* isolates was performed by detecting the *coa* gene. The amplicon sizes of the *23S rRNA*, *nuc*, and *coa* genes were approximately 1250, 279, and 204 bp, respectively (Figure 2). All *S. aureus* isolates from dogs, rabbits, and goat milk were positive for the *coa* gene, whereas 95.8% (23/24) of bovine milk isolates and 72.2% (13/18) of cat isolates were positive.

### Antimicrobial resistance profiles

All *S. aureus* isolates confirmed by phenotypic and genotypic tests were evaluated for antimicrobial susceptibility using the Kirby–Bauer disk diffusion method with the following antibiotics: tetracycline (TE, 30 µg), gentamicin (CN, 10 µg), erythromycin (E, 15 µg), penicillin G (P, 10 µg), cefoxitin (FOX, 30 µg), ciprofloxacin (CIP, 5 µg), and clindamycin (DA, 10 µg).

**Figure 3:**
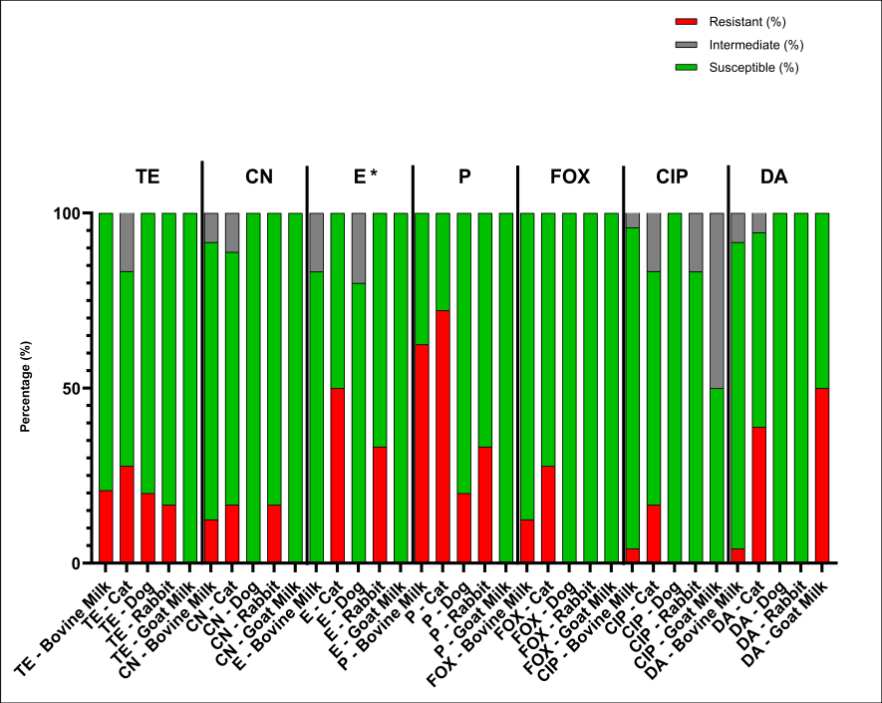
Antimicrobial susceptibility profiles of *Staphylococcus aureus* isolates from bovine milk, cats, dogs, rabbits, and goat milk. TE (tetracycline, 30 micrograms), CN (gentamicin, 10 micrograms), E (erythromycin, 15 micrograms), P (penicillin G, 10 international units), FOX (cefoxitin, 30 micrograms), CIP (ciprofloxacin, 5 micrograms), and DA (clindamycin, 10 micrograms). Differences among animal sources were analyzed using Fisher’s exact test; * = p < 0.05 (significant).

Table 3 and Figure 3 summarize the overall resistance rates among *S. aureus* isolates from various animal sources. The isolates exhibited variable resistance patterns, with the highest rates of resistance to penicillin (20%–72%) and tetracycline (17%–28%), whereas most isolates remained susceptible to ciprofloxacin and cefoxitin. No significant differences in the distribution of antibiotic resistance were observed among animal sources (Fisher’s exact test, p > 0.05). In contrast, erythromycin showed a statistically significant difference (Fisher’s exact test, p < 0.05).

**Table 3:** Antibiotic resistance profiles of *Staphylococcus aureus* isolates isolated from bovine milk, cats, dogs, rabbits, and goat milk in Indonesia.

Antibiotic	Bovine milk (n = 24)	Cats (n = 18)	Dogs (n = 5)	Rabbits (n = 6)	Goat milk (n = 2)
Resistant (%)					
TE (30 µg)	20.8 (5/24)	27.8 (5/18)	20 (1/5)	16.7 (1/6)	0 (0/2)
CN (10 µg)	12.5 (3/24)	16.7 (3/18)	0 (0/5)	16.7 (1/6)	0 (0/2)
E (15 µg)	0 (0/24)	50 (9/18)	0 (0/5)	33.3 (2/6)	0 (0/2)
P (10 µg)	62.5 (15/24)	72.2 (13/18)	20 (1/5)	33.3 (2/6)	0 (0/2)
FOX (30 µg)	12.5 (3/24)	27.8 (5/18)	0 (0/5)	0 (0/6)	0 (0/2)
CIP (5 µg)	4.2 (1/24)	16.7 (3/18)	0 (0/5)	0 (0/6)	0 (0/2)
DA (10 µg)	4.2 (1/24)	38.9 (7/18)	0 (0/5)	0 (0/6)	50 (1/2)
Susceptible (%)					
TE (30 µg)	79.2 (19/24)	55.6 (10/18)	80 (4/5)	83.3 (5/6)	100 (2/2)
CN (10 µg)	79.2 (19/24)	72.2 (13/18)	100 (5/5)	83.3 (5/6)	100 (2/2)
E (15 µg)	83.3 (20/24)	50 (9/18)	80 (4/5)	66.7 (4/6)	100 (2/2)
P (10 µg)	37.5 (9/24)	27.8 (5/18)	80 (4/5)	66.7 (4/6)	100 (2/2)
FOX (30 µg)	87.5 (21/24)	72.2 (13/18)	100 (5/5)	100 (6/6)	100 (2/2)
CIP (5 µg)	91.7 (22/24)	66.7 (12/18)	100 (5/5)	83.3 (5/6)	50 (1/2)
DA (10 µg)	87.5 (21/24)	55.6 (10/18)	100 (5/5)	100 (6/6)	50 (1/2)

TE = Tetracycline, CN = Gentamicin, E = Erythromycin, P = Penicillin G, FOX = Cefoxitin, CIP = Ciprofloxacin, DA = Clindamycin.

### Detection of antibiotic resistance genes

**Figure 4:**
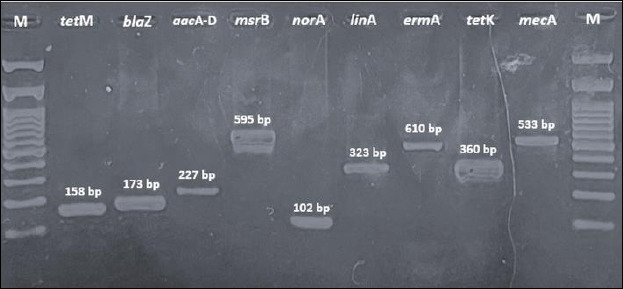
Polymerase chain reaction amplification of antibiotic resistance–encoding genes in *Staphylococcus aureus* isolates from different animal sources. Amplicon sizes: *tetM* (158 base pairs), *blaZ* (173 base pairs), *aacA-D* (227 base pairs), *msrB* (595 base pairs), *norA* (102 base pairs), *linA* (323 base pairs), *ermA* (610 base pairs), *tetK* (360 base pairs), and *mecA* (533 base pairs). M = deoxyribonucleic acid ladder (100 base pairs).

**Figure 5:**
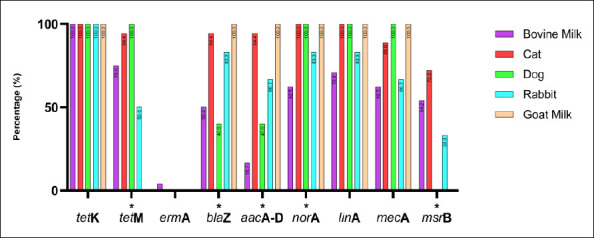
Distribution of antimicrobial resistance genes among *Staphylococcus aureus* isolates from bovine milk, cats, dogs, rabbits, and goat milk in Indonesia. Bars represent gene prevalence by host. Fisher’s exact test was applied; genes detected in all hosts (e.g., *tetK*) were excluded. * = p < 0.05 (significant).

All 55 *S. aureus* isolates from bovine milk, cats, dogs, rabbits, and goat milk were screened for antibiotic resistance–encoding genes using PCR. The genes analyzed were *tetK*, *tetM*, *ermA*, *blaZ*, *aacA-D*, *norA*, *linA*, *mecA*, and *msrB*. Overall, *tetK* was detected in all isolates across all host species, indicating widespread tetracycline resistance. High detection frequencies were also observed for *blaZ*, *norA*, *linA*, and *mecA*, although prevalence varied among animal sources. In contrast, *ermA* and *msrB* were detected at relatively low frequencies and exhibited host-specific distribution patterns. Table 4 summarizes the detailed detection rates by host species, while Figures 4 and 5 show representative PCR amplicons and comparative visualizations of resistance gene profiles. Significant differences were observed for *tetM*, *blaZ*, *aacA-D*, *norA*, and *msrB* (p < 0.05), whereas no significant differences were observed for *ermA*, *linA*, and *mecA*. The *tetK* gene was excluded because it was detected uniformly across all sources.

### Phenotypic and genotypic patterns of multidrug resistance

**Figure 6:**
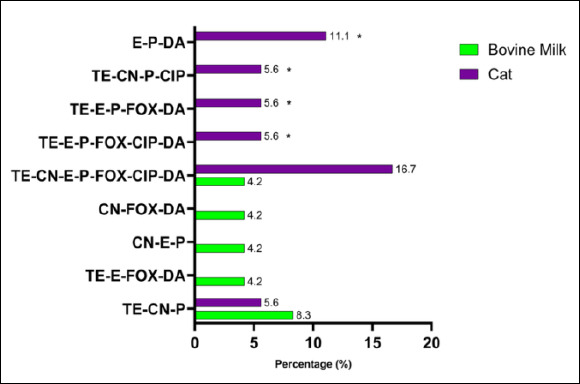
Phenotypic multidrug resistance patterns among *Staphylococcus aureus* isolates from different animal sources in Indonesia. Bars show the percentage of isolates resistant to antibiotic combinations (tetracycline, oxacillin, gentamicin, erythromycin, ampicillin, penicillin, cefoxitin, ciprofloxacin, amoxicillin, and clindamycin). Differences between animal sources were assessed using Fisher’s exact test; * = p < 0.05 (significant).

**Figure 7:**
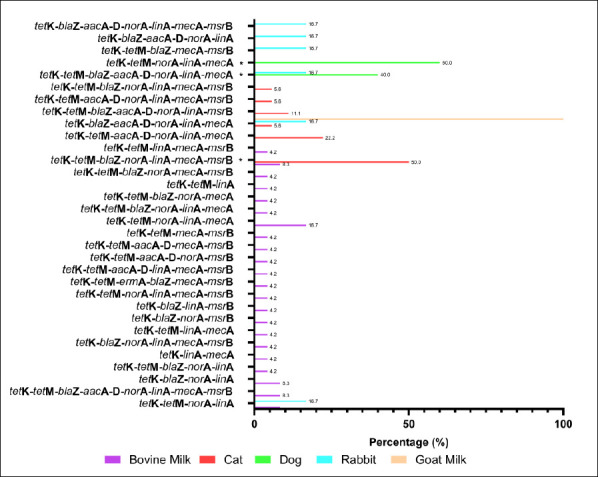
Genotypic multidrug resistance (MDR) profiles of *Staphylococcus aureus* isolates from different animal sources in Indonesia. Bars indicate the percentage of isolates carrying each combination of resistance genes. Fisher’s exact test was applied to MDR profiles with a sufficient frequency; rare profiles were excluded. * = p < 0.05 (significant).

The MDR pattern of *S. aureus* isolates varied by animal source. The most frequent phenotypic combinations included resistance to β-lactams (penicillin, oxacillin, ampicillin, amoxicillin, and cefoxitin), macrolides (erythromycin), and tetracycline (Figure 6). Although several MDR patterns were more common in specific animal sources, none of the observed differences reached statistical significance using Fisher’s exact test. The molecular detection of resistance genes revealed diverse genotypic profiles that matched the observed phenotypes (Figure 7). Several MDR patterns were observed among *S. aureus* isolates from different animal sources, with certain patterns showing significantly higher prevalence in specific sources, particularly dogs and cats. In contrast, the majority of cases occurred at low frequencies and did not differ statistically (p < 0.05). The coexistence of multiple resistance genes was common among *S. aureus* isolates from all animal sources and was dominated by combinations involving *tetK*, often accompanied by *tetM*, *norA*, *linA*, and *mecA*. Overall, gene profiles with ≥ 4 resistance genes accounted for the majority of MDR isolates, with combinations including *tetK*–*tetM* and *tetK*–*norA*–*linA* observed in more than 50% of isolates across host species. More complex gene constellations (≥ 6 genes) were particularly frequent in bovine milk and companion animal isolates, underscoring extensive MDR at the genotypic level. The presence of multiple resistance genes in several isolates indicated the potential for MDR among circulating strains across animal sources.

## DISCUSSION

### Identification and phenotypic variations of *S. aureus*

Based on the biochemical characteristics and PCR amplification of the *23S rRNA* and *nuc* genes, a total of 55 isolates from bovine milk, cats, dogs, rabbits, and goat milk were confirmed to be *S. aureus*. The *23S rRNA* and *nuc* genes are widely accepted molecular markers for *S. aureus* identification [9], whereas the *coa* gene provides additional strain-level specificity [22]. However, several isolates showed discordance between molecular detection of the *coa* gene and phenotypic coagulase testing. Similar inconsistencies have been reported in regional and international studies, indicating that phenotypic coagulase tests alone may underestimate the prevalence of *S. aureus*, particularly in animal-derived isolates, and should be complemented with molecular confirmation [23–25]. Mutations affecting *coa* expression or regulation have been proposed as underlying mechanisms for such discrepancies [24, 26–27].

A small proportion of isolates from bovine milk, cats, dogs, and rabbits were catalase-negative, a rare phenotype in *S. aureus*. Catalase-negative *S. aureus* (CNSA) strains have been linked to *katA* gene mutations. Although they are increasingly reported in both human and veterinary settings, their potential for virulence remains unclear [28]. Their presence in animal sources underscores the importance of molecular diagnostics in preventing misidentification and underreporting.

### Hemolytic activity and host adaptation

Hemolytic activity varies by host origin, reflecting host-adaptive virulence traits. Beta-hemolysis predominated among isolates from cats, consistent with studies implicating β-hemolysin in skin and soft-tissue infections in companion animals [29, 30]. In contrast, gamma-hemolysis was more common among bovine milk isolates, consistent with reports of mastitis-associated *S. aureus* strains in Southeast Asia and elsewhere, where non-hemolytic phenotypes are frequently observed [31–34]. These findings support the concept that host niche and infection ecology influence hemolysin expression.

### Small-colony variants and their implications

In this study, five isolates exhibited atypical growth, forming pinpoint-sized colonies smaller than those of wild-type *S. aureus* (Figure 1), resembling SCVs. SCVs are a subpopulation of *S. aureus* characterized by phenotypic alterations that enable persistence under adverse environmental conditions. SCVs have been increasingly reported among livestock- and companion-animal-associated *S. aureus*, particularly in the context of chronic infections and prolonged antimicrobial exposure [35]. SCVs exhibit enhanced biofilm formation, altered metabolism, and increased antibiotic tolerance, thereby facilitating persistence within the host and environment [36, 37]. Although SCVs were not explicitly characterized in this study, their associations with biofilm formation, intracellular persistence, and antimicrobial tolerance are highly relevant in veterinary settings. SCVs may contribute to chronic or recurrent infections in animals and complicate treatment outcomes, particularly in the context of MDR *S. aureus* [35]. The interplay among methicillin resistance, biofilm production, and host adaptation highlights MRSA as a significant One Health concern, facilitating transmission among animals, humans, and the environment and complicating infection control efforts. The recognition of these phenotypes underscores the need for further investigation into adaptive survival strategies of *S. aureus* in animal sources and their implications for long-term infection control.

### Antimicrobial susceptibility patterns

In this study, the antimicrobial susceptibility profiles showed that resistance patterns were broadly comparable across animal sources for most antibiotics tested, with no statistically significant differences. This suggests a relatively uniform distribution of resistance phenotypes across animal sources, which may reflect similar antimicrobial exposure or shared environmental and management conditions. Erythromycin was the only antimicrobial for which a significant difference was observed among animal sources. Resistance to macrolides may be influenced by host-associated factors or differences in usage patterns. The majority of macrolide use was in food-producing animals; in the U. S. in 2017, macrolides were the most commonly used in cattle [38].

In this study, *S. aureus* isolates from different animal sources exhibited variable resistance patterns, consistent with regional and global reports on antimicrobial resistance in *S. aureus* from livestock and companion animals [39–42]. These differences largely reflect geographic variation in resistance patterns shaped by antimicrobial use practices, regulatory frameworks, and animal production systems. The widespread empirical use of penicillins, tetracyclines, and aminoglycosides in food-producing animals has been identified as a major driver of the emergence of resistance in Indonesia and other Southeast Asian countries [42–45]. This study extends previous Indonesian reports on *S. aureus* by providing a comparative, multi-host analysis of resistance gene profiles across livestock and companion animals within the same geographic regions. Unlike earlier studies that focused on single host species or on phenotypic resistance alone [8, 9, 25, 33], our findings demonstrate shared resistance gene patterns across animal sources, supporting the existence of interconnected AMR reservoirs in a One Health context.

### Multidrug resistance patterns

Bacteria were classified as MDR if they showed resistance or intermediate resistance to at least one antibiotic from three or more antimicrobial classes [12, 13]. MDR *S. aureus* strains were primarily detected in bovine milk and cat isolates (Figure 6). The consistent statistical significance across multiple MDR patterns reflects a structured distribution of resistance combinations rather than independent random events. Many MDR patterns share common antibiotics, resulting in correlated resistance profiles that manifest as significant global associations across multiple combinations. The occurrence of MDR strains in dairy cattle has been strongly associated with repeated antibiotic use for mastitis management, suboptimal milking hygiene, and environmental contamination [42]. Similar trends have been reported in major dairy-producing regions across Asia, underscoring the zoonotic risk posed by MDR *S. aureus* throughout the food chain [45]. Close contact with humans and frequent veterinary interventions in companion animals further increase the potential for interspecies transmission.

MDR *S. aureus* isolates were confirmed by the detection of several antibiotic resistance genes (Figure 7). The frequent co-occurrence of resistance genes, such as *mecA*, *blaZ*, and *tetK*, suggests a potential role for horizontal gene transfer. However, the underlying genetic platforms (e.g., plasmids or SCCmec types) remain uncharacterized and warrant further investigation using whole-genome approaches. Similar findings have been reported in several countries, indicating that MDR *S. aureus* poses a significant challenge to both animal and public health due to its zoonotic potential [45, 46]. A comparison with studies from Malaysia [45] indicates broadly similar resistance trends, reinforcing the regional relevance of our findings. MDR transmission can occur within veterinary clinics through contact among personnel, animals, and owners and may contribute to nosocomial spread or localized outbreaks. The predominance of shared gene combinations across animal hosts further indicates interconnected resistance reservoirs consistent with the One Health framework. Therefore, continuous surveillance and prudent antibiotic use are essential to minimize the emergence and dissemination of MDR *S. aureus* in both livestock and companion animals.

### Phenotypic-genotypic discrepancies

PCR amplification detected several antibiotic resistance genes, including *tetK*, *tetM*, *ermA*, *blaZ*, *aacA-D*, *norA*, *linA*, *mecA*, and *msrB*, in *S. aureus* isolates from diverse animal sources. Genotypic profiles were compared with phenotypic susceptibility results obtained using the Kirby–Bauer disk diffusion method. Discrepancies between resistance genotypes and phenotypes were observed among *S. aureus* isolates obtained from different animal sources. In several cases, isolates exhibited phenotypic resistance despite the absence of corresponding resistance genes. Some isolates phenotypically resistant to oxacillin were *mecA*-negative. This discrepancy may reflect the presence of *mecC*, an analog of *mecA* located on the SCCmec type XI element, which encodes a penicillin-binding protein (PBP2c) that confers β-lactam resistance but has distinct structural and thermosensitive properties [47–50]. Similarly, erythromycin-resistant isolates lacking *ermA* suggest the involvement of other macrolide resistance determinants, particularly *ermC* [51, 52]. The *aacA-D*-negative isolates could also exhibit adaptive resistance [53].

Conversely, several isolates harbored resistance genes but remained phenotypically susceptible. *MecA*-positive but oxacillin-susceptible isolates may be explained by insufficient PBP2a expression or compensatory native PBP activity [54]. The lack of phenotypic β-lactam resistance in some *blaZ*-positive isolates, as well as susceptibility in isolates carrying *tetK*, *tetM*, *aacA-D*, *linA*, *norA*, or *msrB*, is consistent with previous reports describing gene silencing, regulatory suppression, sequence variation, or gene activation [55–59]. In addition, the presence of the *blaZ* gene did not always indicate β-lactam susceptibility, as *mecA* may also mediate resistance [55]. The phenotypic susceptibility of some *linA*-positive isolates may result from mutations in the 50S ribosomal subunit that permit antibiotic binding [57]. Similarly, *norA* detection in ciprofloxacin-susceptible isolates may reflect sequence variation or modulation by global regulators such as *MgrA* [58]. Finally, the discordance between *msrB* detection and erythromycin susceptibility may be attributable to gene mutations or functional overlap with other MLSB resistance determinants, including the erm and *linA* genes [59].

### Factors contributing to MDR emergence

The overall MDR genotypic pattern (Figure 7) corresponded well with the phenotypic resistance profiles. MDR *S. aureus* was detected across multiple animal sources, particularly in isolates from bovine milk and cats. The emergence of MDR strains is strongly linked to inappropriate and excessive antibiotic use in both livestock and companion animals. In veterinary practice, commonly used antimicrobial classes include fluoroquinolones, aminopenicillins (with or without β-lactamase inhibitors), cephalosporins, tetracyclines, sulfonamides, macro-lides, and glycopeptides for the treatment of bacterial infections [60]. In many regions, antibiotic administration often occurs without laboratory diagnosis, adherence to withdrawal periods, or veterinary prescription, as reported in Rwanda, where over 85% of treatments were empirical or extra-label [61]. These practices significantly contribute to the selection and spread of MDR *S. aureus* strains. The detection of multiple resistance genes in phenotypically susceptible isolates suggests the presence of latent resistance reservoirs that may be activated under selective pressure from antimicrobials. Silent or low-expression genes represent an important ecological risk, particularly in environments with frequent or inappropriate antibiotic exposure. The shared distribution of resistance genes across livestock and companion animals likely reflects overlapping ecological niches and common selective pressures, reinforcing the role of animals as interconnected reservoirs of AMR within a One Health framework.

## CONCLUSION

This study provides the first comparative insight into the resistance gene profiles of MDR *S. aureus* isolated from companion and livestock animals in Indonesia. Key results indicate that 55 (45.5%) of 121 isolates were confirmed as *S. aureus*, with the highest prevalence in bovine milk (80%) and rabbits (85.7%). All isolates exhibited MDR phenotypes, predominantly to penicillin (20%–72%), tetracycline (17%–28%), and clindamycin, with significant variation in erythromycin resistance across sources (p < 0.05). Genotypically, *tetK* was ubiquitous (100%), followed by *linA* (85.5%), *norA* (81.8%), *mecA* (76.4%), and *blaZ* (69.1%), with co-occurrence of *mecA*, *blaZ*, and *tetK* suggesting horizontal gene transfer. Phenotypic-genotypic discrepancies were observed, potentially due to alternative mechanisms or regulatory factors, and complex gene combinations (≥4 genes) were present in over 50% of isolates.

The practical implications of these findings underscore the zoonotic potential of MDR *S. aureus* in Indonesia, where close human–animal interactions facilitate interspecies transmission. This highlights the need for integrated AMR surveillance and stewardship programs in veterinary practices, including prudent antibiotic use, improved hygiene in dairy farms, and routine screening in companion animal clinics to mitigate public health risks within a One Health framework.

A major strength of this research lies in its multi-host approach, combining phenotypic and targeted PCR analyses across diverse animal sources, thereby providing a comprehensive baseline for AMR patterns in understudied regions, such as Yogyakarta and Central Java.

Limitations include reliance on targeted PCR, which may miss the full resistome or genetic contexts; uneven sample sizes across species, potentially affecting generalizability; and the absence of human isolates to directly evaluate zoonotic transmission.

Future scope should encompass whole-genome sequencing for detailed resistome mapping, investigation of biofilm and virulence factors, and longitudinal studies on transmission dynamics between animals, humans, and the environment to inform targeted interventions.

In conclusion, these results underscore animals as critical AMR reservoirs and call for collaborative One Health strategies to curb the dissemination of MDR *S. aureus*, ultimately safeguarding animal and human health in Indonesia and beyond.

## DATA AVAILABILITY

All the generated data are included in the manuscript.

## AUTHORS’ CONTRIBUTIONS

ARPY: Performed the experiment, data analysis, and wrote the manuscript. SIO: Conceptualization, funding acquisition, supervision, and wrote the manuscript. GGA: Contributed to sample collection and the experiment. FA: Data analysis and review of the manuscript. All authors have read and approved the final manuscript.

## References

[1] Tang KW, Millar BC, Moore JE (2023). Antimicrobial resistance (AMR). Br J Biomed Sci.

[2] Kariuki S (2024). Global burden of antimicrobial resistance and forecasts to 2050. Lancet.

[3] Djordjevic SP, Jarocki VM, Seemann T, Cummins ML, Watt AE, Drigo B (2024). Genomic surveillance for antimicrobial resistance:a One Health perspective. Nat Rev Genet.

[4] Gilbert W, Thomas LF, Coyne L, Rushton J (2021). Mitigating the risks posed by intensification in livestock production:antimicrobial resistance and zoonoses. Animal.

[5] Ou C, Shang D, Yang J, Chen B, Chang J, Jin F (2020). Prevalence of multidrug-resistant *Staphylococcus aureus* with strong biofilm formation ability in animal-based foods in Shanghai. Food Control.

[6] Widianingrum DC, Windria S, Salasia SI (2016). Antibiotic resistance and methicillin-resistant *Staphylococcus aureus* isolated from bovine, Etawa crossbred goat, and humans. Asian J Anim Vet Adv.

[7] Afshar MF, Zakaria Z, Cheng CH, Ahmad NI (2023). Prevalence and multidrug-resistant profiles of methicillin-resistant *Staphylococcus aureus* and *Staphylococcus pseudintermedius* in dogs, cats, and pet owners in Malaysia. Vet World.

[8] Aziz F, Lestari FB, Nuraidah S, Purwati E, Salasia SI (2016). Detection of genes encoding methicillin, penicillin, and tetracycline resistance in *Staphylococcus aureus* isolated from subclinical mastitis milk. J Sain Vet.

[9] Fitranda M, Salasia SI, Sianipar O, Dewananda DA, Arjana AZ, Aziz F (2023). Methicillin-resistant *Staphylococcus aureus* isolates derived from humans and animals in Yogyakarta, Indonesia. Vet World.

[10] World Health Organization (2015). Global action plan on antimicrobial resistance. https://iris.who.int/handle/10665/193736.

[11] Clinical and Laboratory Standards Institute (2024). Performance standards for antimicrobial susceptibility testing. CLSI supplement M100.

[12] Magiorakos AP, Srinivasan A, Carey RB, Carmeli Y, Falagas ME, Giske CG (2012). Multidrug-resistant, extensively drug-resistant and pandrug-resistant bacteria:an international expert proposal for interim standard definitions for acquired resistance. Clin Microbiol Infect.

[13] De Almeida CC, Pizauro LJ, Soltes GA, Slavic D, De Avila FA, Pizauro JM (2018). Some coagulase-negative *Staphylococcus* spp. isolated from buffalo misidentified as *Staphylococcus aureus* by phenotypic and Sa442 PCR methods. BMC Res Notes.

[14] Quraishi A, Kaur P, Sharma NS, Arora AK (2021). Antibiotic sensitivity patterns of *Staphylococcus* spp. isolated from goat milk with molecular detection of resistance genes. Iran J Vet Res.

[15] Chamgordami AZ, Momtaz H, Zia-Jahromi N (2022). Distribution of antibiotic resistance genes among methicillin-resistant *Staphylococcus aureus* from human infections. Acad J Health Sci.

[16] Khan SA, Nawaz MS, Khan AA, Steele RS, Cerniglia CE (2000). Characterization of erythromycin resistance methylase genes in multidrug-resistant *Staphylococcus* spp. from bovine milk. Am J Vet Res.

[17] Lina G, Quaglia A, Reverdy ME, Leclercq R, Vandenesch F, Etienne J (1999). Distribution of macrolide, lincosamide, and streptogramin resistance genes among staphylococci. Antimicrob Agents Chemother.

[18] Aziz KE, Abdulrahman ZF (2021). Detection of tetracycline resistance gene tet(K) in clinical *Staphylococcus aureus* isolates. IOP Conf Ser Earth Environ Sci.

[19] Saengsawang P, Tanonkaew R, Kimseng R, Nissapatorn V, Wintachai P, Rodriguez-Ortega MJ (2025). Whole-genome analysis of multidrug-resistant *Staphylococcus aureus* and *Staphylococcus pseudintermedius* from dogs and cats. Antibiotics (Basel).

[20] Di Francesco CE, Smoglica C, Profeta F, Farooq M, Di Giannatale E, Toscani T (2021). Detection of antibiotic resistance genes in commercial poultry and turkey flocks. Poult Sci.

[21] Hamady AB, El-Fadeal AN, Imbaby S, Nassar HM, Sakr MG, Marei YE (2024). Expression of *norA*, *norB*, and *norC* efflux pump genes in MRSA isolates. J Infect Dev Ctries.

[22] Veras JF, do Carmo LS, Tong LC, Shupp JW, Cummings C, dos Santos DA (2008). Enterotoxigenicity of coagulase-positive and -negative staphylococci from food poisoning outbreaks. Int J Infect Dis.

[23] Sunagar R, Deore SN, Deshpande PV, Rizwan A, Sannejal AD, Sundareshan S (2013). Differentiation of *Staphylococcus aureus* and *Staphylococcus epidermidis* by PCR for the fibrinogen-binding protein gene. J Dairy Sci.

[24] Younis G, Sadat A, Maghawry M (2018). Characterization of coa gene and antimicrobial profiles of S*taphylococcus aureus* isolated from bovine clinical and subclinical mastitis. Adv Anim Vet Sci.

[25] Effendi MH, Hisyam MA, Hastutiek P, Tyasningsih W (2019). Detection of coagulase gene in *Staphylococcus aureus* from several dairy farms in East Java, Indonesia, by polymerase chain reaction. Vet World.

[26] Phonimdaeng P, O'Reilly M, Nowlan P, Bramley AJ, Foster TJ (1990). The coagulase of *Staphylococcus aureus* 8325-4:sequence analysis and virulence of site-specific coagulase-deficient mutants. Mol Microbiol.

[27] Valmorbida MK, Cardozo MV, Almeida CC, Pereira N, Dezen D, Assis MZ (2022). Association between *coa* gene and enterotoxin gene in Staphylococcus aureus from dairy cattle in Brazil. Food Sci Technol.

[28] Laub K, Kristof K, Tirczka T, Tothpal A, Kardos S, Kovacs E (2017). First description of a catalase-negative *Staphylococcus aureus* from a healthy carrier with a novel nonsense mutation in the *katA* gene. Int J Med Microbiol.

[29] Pontieri E (2018). The staphylococcal hemolysins. Pet-to-Man Travelling Staphylococci.

[30] Al-Saraj MN, Abdullah AH (2020). Bacteriological study of *Staphylococcus aureus* isolated from skin-infected sheep. Plant Arch.

[31] Shrivastava N, Sharma V, Shrivastav A, Nayak A, Rai AK (2018). Prevalence and characterization of Panton–Valentine leukocidin-positive *Staphylococcus aureus* in bovine milk in Jabalpur district of Madhya Pradesh, India. Vet World.

[32] Laukova A, Bino E, Kubasova I, Strompfova V, Simonova MP (2021). Variability in fecal canine staphylococci identified by MALDI-TOF mass spectrometry, their properties and susceptibility to bacteriocins. EC Microbiol.

[33] Salasia SI, Khusnan C, Lämmler C, Zschöck M (2004). Comparative studies on pheno- and genotypic properties of *Staphylococcus aureus*, isolated from bovine subclinical mastitis in Central Java in Indonesia and Hesse in Germany. J Vet Sci.

[34] Ariyanti D, Salasia SI, Tato S (2011). Characterization of haemolysin of *Staphylococcus aureus* isolated from food animal origin. Indones J Biotechnol.

[35] Silva V, Correia E, Pereira JE, González-Machado C, Capita R, Alonso-Calleja C (2022). Biofilm formation of *Staphylococcus aureus* from pets, livestock, and wild animals:relationship with clonal lineages and antimicrobial resistance. Antibiotics (Basel).

[36] Keim KC, George IK, Reynolds L, Smith AC (2023). The clinical significance of *Staphylococcus aureus* small colony variants. Lab Med.

[37] Aboelnaga N, Elsayed SW, Abdelsalam NA, Salem S, Saif NA, Elsayed M (2024). Deciphering the dynamics of methicillin-resistant *Staphylococcus aureus* biofilm formation:from molecular signaling to nanotherapeutic advances. Cell Commun Signal.

[38] Trott DJ, Turnidge J, Kovac JH, Simjee S, Wilson D, Watts J (2021). Comparative macrolide use in humans and animals:should macrolides be moved off the World Health Organisation's critically important antimicrobial list?. J Antimicrob Chemother.

[39] Lubna HT, Shami A, Rafiq N, Khan S, Kabir M, Khan NU (2023). Antimicrobial usage and detection of multidrug-resistant *Staphylococcus aureus*:methicillin- and tetracycline-resistant strains in raw milk of lactating dairy cattle. Antibiotics (Basel).

[40] Ceniti C, Britti D, Santoro AM, Musarella R, Ciambrone L, Casalinuovo F (2017). Phenotypic antimicrobial resistance profile of isolates causing clinical mastitis in dairy animals. Ital J Food Saf.

[41] Abdullahi IN, Zarazaga M, Campaña-Burguet A, Eguizábal P, Lozano C, Torres C (2022). Nasal *Staphylococcus aureus* and *S. pseudintermedius* carriage in healthy dogs and cats:a systematic review of their antibiotic resistance, virulence and genetic lineages of zoonotic relevance. J Appl Microbiol.

[42] Khairullah AR, Sudjarwo SA, Effendi MH, Ramandinianto SC, Gelolodo MA, Widodo A (2022). Profile of multidrug resistance and methicillin-resistant *Staphylococcus aureus* (MRSA) on dairy cows and risk factors from farmer. Biodiversitas.

[43] Lianou DT, Fthenakis GC (2022). Use of antibiotics against bacterial infections on dairy sheep and goat farms:patterns of usage and associations with health management and human resources. Antibiotics (Basel).

[44] Virto M, Santamarina-García G, Amores G, Hernández I (2022). Antibiotics in dairy production:where is the problem?. Dairy.

[45] Haulisah NA, Hassan L, Jajere SM, Ahmad NI, Bejo SK (2022). High prevalence of antimicrobial resistance and multidrug resistance among bacterial isolates from diseased pets:retrospective laboratory data (2015–2017). PLoS One.

[46] Kaspar U, von Lützau A, Schlattmann A, Roesler U, Köck R, Becker K (2018). Zoonotic multidrug-resistant microorganisms among small companion animals in Germany. PLoS One.

[47] García-Álvarez L, Holden MT, Lindsay H, Webb CR, Brown DF, Curran MD (2011). Methicillin-resistant *Staphylococcus aureus* with a novel *mecA* homologue in human and bovine populations in the UK and Denmark:a descriptive study. Lancet Infect Dis.

[48] Shore AC, Deasy EC, Slickers P, Brennan G, O'Connell B, Monecke S (2011). Detection of staphylococcal cassette chromosome mec type XI carrying highly divergent *mecA*, *mecI*, *mecR1*, *blaZ*, and *ccr* genes in human clinical isolates of clonal complex 130 methicillin-resistant *Staphylococcus aureus*. Antimicrob Agents Chemother.

[49] Ballhausen B, Kriegeskorte A, Schleimer N, Peters G, Becker K (2014). The *mecA* homolog *mecC* confers resistance against β-lactams in *Staphylococcus aureus* irrespective of the genetic strain background. Antimicrob Agents Chemother.

[50] Aqib AI, Ijaz M, Farooqi SH, Ahmed R, Shoaib M, Ali MM (2018). Emerging discrepancies in conventional and molecular epidemiology of methicillin-resistant *Staphylococcus aureus* isolated from bovine milk. Microb Pathog.

[51] Talebi G, Hashemia A, Goudarzi H, Shariati A, Bostanghadiri N, Sharahi JY (2019). Survey of *ermA*, *ermB*, *ermC* and *mecA* genes among *Staphylococcus aureu*s isolates isolated from patients admitted to hospitals in Tehran, Iran by PCR and sequencing. Biomed Res.

[52] Rajkumar S, Sistla S, Manoharan M, Nagasundaram N, Parija SC, Ray P (2017). Prevalence and genetic mechanisms of antimicrobial resistance in *Staphylococcus* species:a multicentre report of the Indian Council of Medical Research antimicrobial resistance surveillance network. Indian J Med Microbiol.

[53] Rasheed H, Ijaz M, Ahmed A, Javed MU, Shah SF, Anwaar F (2023). Discrepancies between phenotypic and genotypic identification methods of antibiotic-resistant genes harbouring *Staphylococcus aureus*. Microb Pathog.

[54] Bressler AM, Williams T, Culler EE, Zhu W, Lonsway D, Patel JB (2005). Correlation of penicillin binding protein 2a detection with oxacillin resistance in *Staphylococcus aureus* and discovery of a novel penicillin binding protein 2a mutation. J Clin Microbiol.

[55] Ferreira C, Abrantes P, Costa SS, Viveiros M, Couto I (2022). Occurrence and variability of the efflux pump gene norA across the *Staphylococcus* genus. Int J Mol Sci.

[56] Emaneini M, Bigverdi R, Kalantar D, Soroush S, Jabalameli F, Noorazar KB (2013). Distribution of genes encoding tetracycline resistance and aminoglycoside-modifying enzymes in *Staphylococcus aureus* strains isolated from a burn centre. Ann Burns Fire Disasters.

[57] Garneau P, Labrecque O, Maynard C, Messier S, Masson L, Archambault M (2010). Use of a bacterial antimicrobial resistance gene microarray for the identification of resistant *Staphylococcus aureus*. Zoonoses Public Health.

[58] Uddin MJ, Ahn J (2017). Associations between resistance phenotype and gene expression in response to serial exposure to oxacillin and ciprofloxacin in *Staphylococcus aureus*. Lett Appl Microbiol.

[59] Romsang A, Atichartpongkul S, Trinachartvanit W, Vattanaviboon P, Mongkolsuk S (2013). Gene expression and physiological role of *Pseudomonas aeruginosa* methionine sulfoxide reductases during oxidative stress. J Bacteriol.

[60] Caneschi A, Bardhi A, Barbarossa A, Zaghini A (2023). The use of antibiotics and antimicrobial resistance in veterinary medicine, a complex phenomenon:a narrative review. Antibiotics (Basel).

[61] Iraguha B, Mpatswenumugabo JP, Gasana MN, Åsbjer E (2024). Mitigating antibiotic misuse in dairy farming systems and milk value chain market:insights into practices, factors, and farmers education in Nyabihu district, Rwanda. One Health.

